# Root-associated fungi in acid mine drainage-impacted environments

**DOI:** 10.3389/fmicb.2026.1812818

**Published:** 2026-06-10

**Authors:** Karianne Forcier, Damase P. Khasa, Sophie Turcotte, Bérenger Bourgeois, Chantal J. Beauchamp

**Affiliations:** 1 Département de Phytologie and Centre Sève, Faculté des Sciences de l'agriculture et de l'alimentation, Pavillon Paul-Comtois, Université Laval, Québec City, QC, Canada; 2 Centre for Forest Research and Institute of Integrative and Systems Biology, Université Laval, Quebec City, QC, Canada; 3 Mining Site Restoration Branch, Ministry of Natural Resources and Forests, Val d’Or, QC, Canada; 4 Québec Centre for Biodiversity Science, Department of Biology, McGill University, Montreal, QC, Canada

**Keywords:** acid mine tailings, *Cadophora*, dark septate endophyte, fungi-assisted remediation, *Phialocephala*, *Populus balsamifera*, *Scirpus atrocinctus*, *Typha latifolia*

## Abstract

Acid mine drainage (AMD) poses persistent environmental challenges despite ongoing restoration efforts, particularly through the generation of acidic and metal-rich substrates. Fungi-assisted phytoremediation has emerged as a complementary strategy to support plant establishment in these environments. This study examines culturable root-associated fungi from four plant species, *Populus balsamifera* (PBA), *Salix discolor* (SDI), *Scirpus atrocinctus* (SAT), and *Typha latifolia* (TLA), growing at an AMD-impacted site and a nearby non-impacted reference site. Culture-dependent estimates of culturable fungi were obtained from bulk soil, rhizosphere soil, and root samples using two culture media. The taxonomic composition of culturable root-associated fungi was characterized, and relationships among taxa, soil physicochemical properties, and plant mineral parameters were examined. Estimates of culturable fungi on Modified Melin-Norkrans medium increased from bulk soil to roots, indicating stronger associations with root compartments under AMD-related stress. Most of the isolates belonged to Ascomycota, with a substantial proportion identified as dark septate endophytes (DSE). *Phialocephala* spp. were strongly associated with SAT and TLA growing in acidic, metal-rich, and water-saturated soils typical of AMD-impacted sites, whereas *Cadophora* spp. were more frequently associated with plants growing under less stressful environmental conditions. Overall, these results highlight the influence of site-specific edaphic conditions on the composition of culturable root-associated fungi and their associations with plant hosts. Together, these patterns suggest that specific fungal-plant associations may contribute to fungi-assisted phytoremediation in AMD-impacted environments.

## 1 Introduction

Acid mine drainage (AMD) arises from the oxidation of sulfide-rich materials exposed during mining activities, generating acidic and metal-rich waters (Rambabu et al., 2020; Singer and Stumm, 1970). This geochemical process can persist long after mine closure, regardless of mitigation efforts. Despite large-scale remediation measures designed to limit oxygen and water infiltration into mine tailings, AMD generation often continues, resulting in pronounced spatial heterogeneity in acidity, metal availability, and hydrology.

In the Canadian province of Quebec, the Aldermac mine represents a remediated yet persistently AMD-impacted environment (Maqsoud et al., 2022). Notably, remediation interventions reshaped the site’s landscape by creating hills, streams, and floodplains. As a consequence, dry upper-slope positions contrast with progressively water-saturated toe-slope environments, where acidic and metal-rich conditions prevail and shape plant establishment. This spatial heterogeneity provides an opportunity to examine how hydrology, soil physicochemical properties, plant mineral dynamics, and belowground microbial filters interact.

Within this hydrological and edaphic environment, phytoremediation has emerged as an approach that relies on the capacity of tolerant plant species to establish in acidic, metal-rich, and hydrologically constrained substrates. However, plant persistence and performance under AMD stress depend on processes operating along the soil-root-shoot gradients to govern mineral uptake and translocation. Plant roots represent a critical interface where elemental uptake begins and where microbial communities are filtered by soil conditions and host plants (Cai et al., 2024; Kalu et al., 2021). Notably, several fungal taxa associated with metal-rich environments have been shown to tolerate high metal loads and interact closely with plant hosts, contributing to plant performance under edaphic stress (Domka et al., 2019; Gagnon et al., 2020; Jumpponen and Trappe, 1998). Under AMD-related stress, this filtering is expected to favor microbial taxa with traits adapted to acidic, metal-rich, and water-saturated soil conditions.

Among belowground microbial groups, fungi represent a key component of root-associated communities in stressed environments (Berthelot et al., 2019). In metal-rich and acidic soils, where both chemical and hydrological conditions vary, shifts in fungal functional groups, including saprotrophs, mycorrhizal fungi, endophytes, and yeasts, have frequently been reported, with dark septate endophytes (DSE) often increasing in relative importance (Domka et al., 2019; Gagnon et al., 2020; Jumpponen and Trappe, 1998). Many root-associated fungi found in such environments display stress-related traits, including melanized hyphae, flexible nutritional strategies, and the capacity to persist under low pH and high metal loads. These functional attributes, rather than taxonomic identity alone, are increasingly recognized as critical for understanding plant-fungal interactions under extreme edaphic conditions.

This study focuses on the culturable fraction of root-associated fungi, acknowledging that culture-dependent methods capture only a subset of total fungal diversity. However, the isolation of culturable species enables further investigation of their ecological functions. Within this framework, the taxonomic composition of culturable root-associated fungi was examined, and the observed patterns were interpreted in terms of ecological guilds and stress-related functional traits inferred from the literature.

Four plant species, *Populus balsamifera, Salix discolor, Scirpus atrocinctus*, and *Typha latifolia* were examined along contrasting topographic and hydrological positions at an AMD-impacted site and a nearby non-impacted reference site. These plant species are widely recognized for their metal tolerance and accumulation capacity and are commonly used in phytoremediation (Khalid et al., 2021; Tőzsér et al., 2023; Wani et al., 2020). Although phytoremediation using these species can contribute to the stabilization of AMD-impacted sites, important knowledge gaps persist regarding mineral and metal bioaccumulation and translocation in impacted compared to nearby non-impacted sites. Therefore, understanding elemental dynamics along the soil–root–shoot continuum is essential for evaluating plant performance and phytoremediation potential under contrasting edaphic stress (Antoniadis et al., 2017).

Accordingly, this study aimed to: (1) compare soil physicochemical properties and plant root and shoot mineral contents across sites and slope positions; (2) estimate the abundance of culturable fungi in bulk soil, rhizosphere soil, and roots using a culture-dependent Most Probable Number (MPN) approach; (3) determine the taxonomic composition of culturable root-associated fungi; and (4) examine the relationships between selected soil and plant mineral parameters and fungal taxa along AMD-related environmental gradients.

By linking culturable fungal assemblages to functional traits and edaphic gradients, this study aims to advance understanding of how root-associated fungi contribute to plant persistence under AMD-impacted ecosystems and to inform the development of fungi-assisted phytoremediation strategies.

## 2 Materials and methods

### 2.1 Site description and sampling

**Figure 1:**
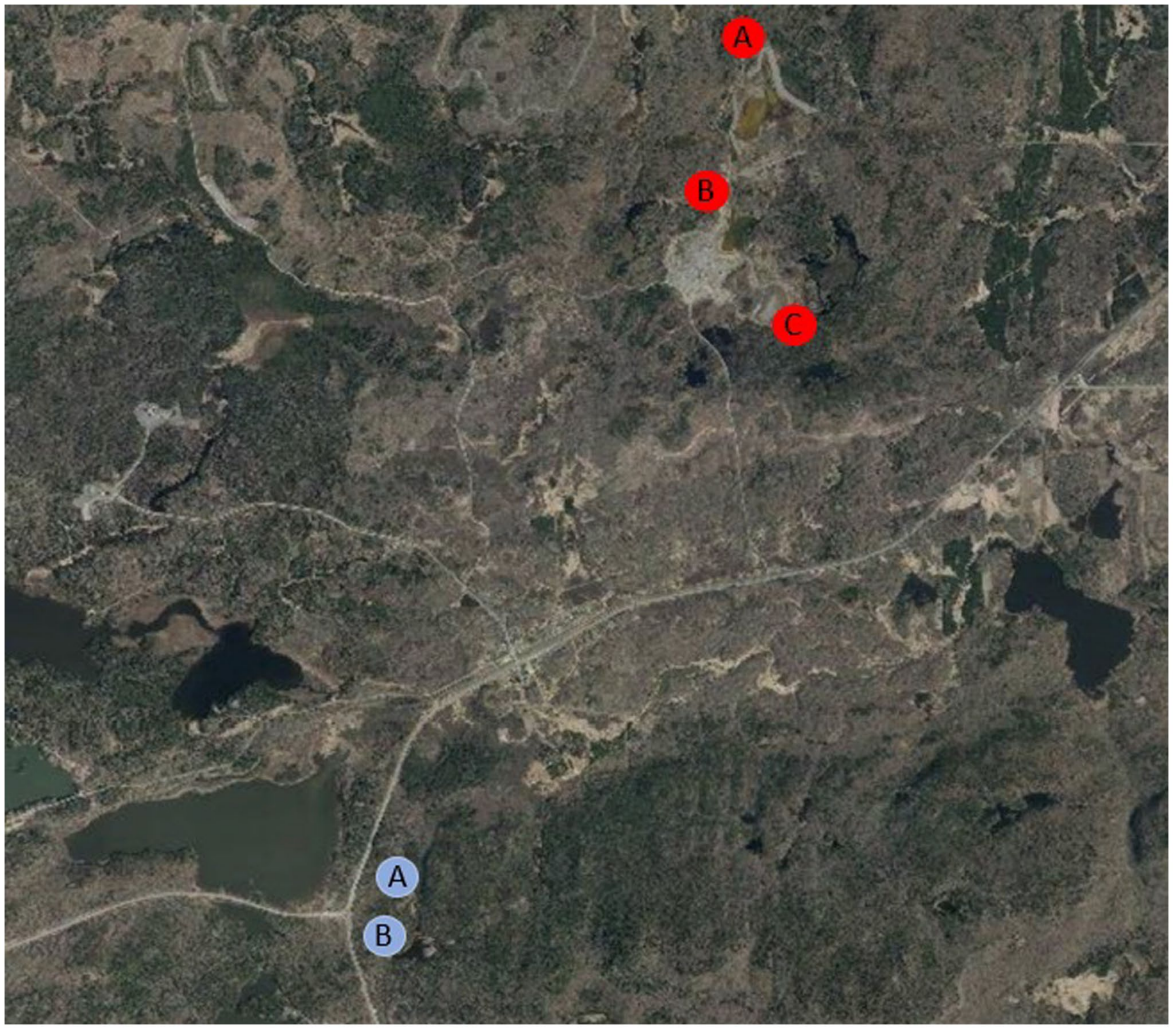
Locations of plant sampling zones at the AMD-impacted site (Aldermac; zones A–C, red) and the non-AMD-impacted site (Kekeko Hills; zones A–B, blue), near Rouyn-Noranda, Quebec, Canada. Zone labels correspond to sample identification numbers reported in Table 1.

This study was conducted in northwestern Quebec, Canada, at the former Aldermac mine site (Barrett et al., 1991). This site was selected because it represents a remediated and revegetated AMD-impacted environment located in the Abitibi region approximately 15 km west of Rouyn*–*Noranda (48.22113° N, −79.23083° W), covering approximately 76 ha and surrounded by forested peatlands and streams (Figure 1). Water-edge zones (i.e., riparian areas along the stream and adjacent pond-edge/littoral margins) affected by AMD exhibited rust-colored sandy soils in lower positions and rocky substrates from the middle to the upper slopes. For comparison, the Kekeko Hills area (48.17583° N, −79.26694° W), located 6 km southwest of the Aldermac site, served as a non-AMD-impacted reference site, allowing the evaluation of soil, plant, and fungal parameters under AMD-impacted and non-impacted conditions. The forested hills included ponds, streams, and lakes with water-edge zones characterized by black muddy soils and mid- to upper-slope positions by clay loam.

Sampling was conducted on the 23rd and 24th of September 2020 following Gagné et al. (2006). At the AMD-impacted site, samples were collected across three zones, whereas two zones were sampled at the non-AMD-impacted site (Figure 1), and each sample was assigned a unique identifier reported in Table 1. The plant species sampled were balsam poplar (*Populus balsamifera;* PBA), glaucous willow (*Salix discolor;* SDI), black-girdled bulrush (*Scirpus atrocinctus*; SAT), and broadleaf cattail (*Typha latifolia;* TLA). At the AMD-impacted site, PBA was collected only from the dry upper-slope zone (*n* = 3). SDI was sampled at the mid-slope (*n* = 3) and toe-slope (*n* = 2), while SAT and TLA were sampled in water-edge zones (*n* = 3 per species). At the non-AMD-impacted site, three samples of each species were collected. Plant heights ranged from 30 to 100 cm. In total, 32 plant samples, including data on aerial parts (stems, shoots, leaves) and roots (roots and fine roots) were collected. The samples were refrigerated at 4 °C and processed within 5 days of collection.

**Table 1:** Replicates per plant species by site, hillslope environment, and sampling zones.

Site	Environment	Zone^1^	Replicates per species^2,3^			
			*Populus balsamifera* (PBA)	*Salix discolor* (SDI)	*Scirpus atrocinctus* (SAT)	*Typha latifolia* (TLA)
AMD-impacted	Toe-slope	A	0	0	3 (9, 10, 11)	3 (18, 19, 20)
	Mid-slope	A	0	2 (7, 8)	0	0
	Toe-slope	B	0	0	3 (12, 13, 14)	3 (15, 16, 17)
	Mid-slope	C	0	3 (4, 5, 6)	0	0
	Upper-slope	C	3 (1, 2, 3)	0	0	0
Non-AMD-impacted	Toe-slope	A	0	0	3 (27, 28, 29)	3 (30, 31, 32)
	Mid-slope	B	0	3 (24, 25, 26)	0	0
	Upper-slope	B	3 (21, 22, 23)	0	0	0

^1^Zones (A-C) correspond to Figure 1.

^2^0 indicates that the species was absent (not sampled) in that zone.

^3^Values in parentheses indicate the sample identification number (sample ID).

### 2.2 Physicochemical analyses of soils and plants

Bulk mineral soil samples were collected approximately 15 cm from plant roots at a depth of 15–20 cm, after removal of the organic layer (the upper 1–2 cm litter). Fresh soil samples were analyzed for water content (WC), pH, and electrical conductivity (EC), according to Conseil des productions végétales du Québec (1997). Soil samples were air dried to constant mass, sieved through a 2-mm mesh, and ground to 60-mesh (Retsch Centrifugal Mill Model ZM-1, Brinkmann Instruments Canada Ltd., Rexdale, ON, Canada). Following soil analyses, the plant material was processed for elemental quantification. Specifically, the plant roots and shoots were cleaned, dried to constant mass at 65 °C, and subsequently ground using a mixer mill (Retsch Mixer Mill Model ZM-2, Brinkmann Instruments, Canada Ltd., Rexdale, ON, Canada).

Elemental contents in the soil, root, and shoot samples were determined by X-ray fluorescence spectrophotometry (Niton™ XL3t Ultra and Niton™ XL3t Gold; Thermo Scientific™) at Université Laval (Jenkins et al., 2025). The elements analyzed were P, K, Ca, S, Cu, Zn, Fe, Al, Si, Mo, Mn, Cr, As, Co, Ni, Pb. For clarity, soil elemental contents are reported using the element symbol alone (e.g., P), whereas root and shoot concentrations are indicated by appending “R” or “S,” respectively (e.g., RP for root phosphorus; SP for shoot phosphorus). The X-ray fluorescence spectrophotometry method was validated by Lamont Inc (2017), demonstrating high reliability with a 95% correlation for metal detection. These characteristics, along with its rapid analysis, reduced cost, broad multi-element capacity, and extended range for metal quantification (Jenkins et al., 2025), made it well suited for the present study.

To quantify element accumulation and mobility within plants, root and shoot bioaccumulation factors (RBCF and SBCF), and translocation factors (TF), were calculated following Antoniadis et al. (2017):


$$\text{RBCF}=\frac{\text{Root content}}{\text{Soil content}},\text{SBCF}=\frac{\text{Shoot content}}{\text{Soil content}},\mathrm{TF}=\frac{\text{Shoot content}}{\text{Root content}}$$

Bioconcentration factor (BCF) values greater than 1 indicate accumulation in plant tissues relative to soil, whereas values lower than 1 indicate limited accumulation. Translocation factor values greater than 1 indicate preferential translocation of elements to shoot, while values lower than 1 indicate retention in root.

### 2.3 Fungal estimation and isolation

Culturable fungi were estimated using the Most Probable Number (MPN) approach (Blodgett, 2020) from three root-associated compartments: bulk soil, rhizosphere soil, and roots. Rhizosphere soil was operationally defined as soil loosely adhered to roots and was obtained by gently shaking roots in a sterile plastic bag. After rhizosphere soil removal, fine roots were surface-disinfected using a brief sterilization protocol consisting of immersion in 95% ethanol for 10 s, followed by 0.6% sodium hypochlorite (NaOCl) supplemented with 20 μL Tween 80 for 30 s, and nine successive rinses in sterile distilled water (Brundrett et al., 1996; McPherson et al., 2018). Subsequently, the root segments were aseptically dried at room temperature and cut into 1–2 cm pieces.

Two complementary isolation methods were used to recover root-associated fungi, targeting potentially overlapping fractions. Because the final rinse water was not plated, both methods were interpreted as overlapping root-associated fungi, rather than fungi strictly located on the root surface or within internal tissues. To achieve this, a portion of the surface-disinfected root pieces was aseptically ground with a sterile mortar and pestle to obtain a fine homogenate for dilution-based MPN estimation, while the remaining pieces were kept intact for direct isolation.

For MPN estimation, three independent replicates (each consisting of 0.1 g dry mass) were prepared for each compartment: bulk soil, rhizosphere soil, and ground-root homogenates. The samples were suspended in 100 mL sterile phosphate-saline buffer and homogenized, followed by tenfold serial dilutions (10^−1^ to 10^−6^). For each dilution, eight 100-μL drops were plated onto two mycorrhizal fungi and dark septate endophytes (DSE) selective culture media: (1) Rose Bengal agar (RBA; Difco, Becton-Dickinson; pH 7.2, supplemented with chloramphenicol 100 mg l^−1^), and (2) Modified Melin-Norkrans medium (MMN) (Brundrett et al., 1996). MMN plates were amended with antibiotics (chlortetracycline 30 mg l^−1^, ampicillin 10 mg l^−1^, streptomycin 80 mg l^−1^) and benomyl 10 mg l^−1^ (methyl [1-(butylcarbamoyl)-1H-benzimidazol-2-yl]carbamate). Plates were incubated at 22 °C ± 2 °C and examined every 1–2 days. Culturable fungi were enumerated on both media, and counts were converted to MPN g dry matter using the 8-drop MPN approach (Blodgett, 2020).

Fungal isolates were obtained from colonies arising either from serial dilutions of the ground-root homogenate (ground-root isolates) or from intact, surface-disinfected root pieces placed on MMN (disinfected-root-piece isolate). The obtained isolates were subcultured repeatedly on MMN until pure cultures were obtained, while for long-term storage, the pure isolates were maintained on potato dextrose agar (PDA) or malt extract agar (MEA).

### 2.4 Molecular identification of fungal isolates

Fungal isolates were identified using a molecular approach based on DNA extraction, ITS amplification, and Sanger sequencing. DNA was extracted using the DNeasy®Plant Mini Kit (QIAGEN, Toronto, ON, Canada). Approximately 50 mg of fungal mycelium was ground in liquid nitrogen using a sterile mortar and pestle and processed according to the manufacturer’s protocol. The internal transcribed spacer (ITS) region was amplified by PCR following Gagné et al. (2006). Each 20 μL PCR reaction contained 2.0 μL buffer, 0.6 μL MgCl_2_, 0.2 μL bovine serum albumin, 0.2 μL dNTPs, 0.16 μL of each primer (ITS1F and ITS4), 0.1 μL Platinum® Taq polymerase (Invitrogen, Carlsbad, CA) and 2 μL of extracted DNA. PCR amplification was performed using a PTS-225 thermocycler (MJ Research, Waltham, MA). The program consisted of an initial denaturation step of 2.5 min at 95 °C, followed by 13 cycles of 30 s at 95 °C, 55 s at 55 °C, and 45 s at 72 °C. This was followed by 12 cycles with an extension time of 2 min at 72 °C, 4 cycles of 3 min at 72 °C, and a final extension of 10 min at 72 °C. Amplification success was verified by electrophoresis on a 1% agarose gel, followed by Sanger sequencing of PCR products at the IBIS genomic analysis platform using the ABI 3130XL genetic analyzer (Applied Biosystems, Foster City, CA).

Sequences were cleaned and paired using Geneious Prime v2021.2.2 software (Biomatters). The high-quality sequence data were subsequently processed into operational taxonomic units (OTUs) for downstream analyses. OTUs were clustered with the CD-HIT-EST web server at 97% global sequence identity. The longest sequence per cluster was retained as the representative. All downstream analyses (counts, taxonomic summaries, and associations) refer to OTUs (not individual sequences). Representative OTU sequences were queried by BLASTn against the GenBank nt database. Species-level names were reported when percent identity was ≥ 99% and percent query coverage was ≥ 98%; otherwise, the genus-level labels (e.g., *Mortierella* sp.) or higher ranks were retained. Species names are thus provisional/*sensu lato* for taxa with limited ITS resolution (e.g., *Fusarium, Mortierella*).

### 2.5 Statistical analyses

All statistical analyses were conducted to evaluate the effects of site, plant species, and associated environmental variables on soil, plant, and fungal parameters. Analyses were performed using SAS OnDemand for Academics (SAS Institute, Cary, NC). Data were assessed for normality and homogeneity of variance, using the Shapiro–Wilk and Levene’s tests, respectively (Montgomery, 2019). When required, data were transformed to meet ANOVA assumptions, and means were reported after back-transformation.

Two-way ANOVA was performed for soil physicochemical properties, plant root and shoot mineral and metal contents, and bioconcentration and translocation factors (BCF and TF), with Site (S; AMD-impacted vs. non-AMD-impacted) and Plant species (P; PBA, SDI, SAT, TLA) as fixed factors. Where significant Site × Plant species (S × P) interactions were observed, means were compared using Scheffé’s test (*p* < 0.05).

Estimates of fungal abundance were analyzed using a four-factor ANOVA with Site, Plant species, Root zone (bulk soil, rhizosphere soil, and roots), and Culture medium (MMN or RB) as fixed factors. Significant interactions, including Site × Root zone × Culture medium, were compared using Scheffé’s test.

Canonical redundancy analysis (RDA) was used to examine relationships among soil physicochemical variables, plant elemental contents, bioconcentration factors (RBCF, SBCF), translocation factors (TF), and fungal genera. RDA and permutation tests were conducted in R using vegan package and graphical output was generated with ggplot2 (RStudio Team, 2020). Explanatory variables were selected by forward-backward stepwise selection based on 999 permutation tests (Venables and Ripley, 2002).

## 3 Results

### 3.1 Physicochemical characteristics of soil and plants

Soil physicochemical properties differed significantly between sites for EC, K, Cu, Mo, Mn, and Pb (*p* < 0.05; Tables 2, 3; Supplementary Tables S1, S2). Across plant species, the AMD-impacted site exhibited significantly higher EC, Cu and Mo contents, whereas K, Pb, and Mn contents were significantly lower than at the non-AMD-impacted site (Supplementary Tables S1, S2). Soil properties also differed significantly among plant species (Table 2). Briefly, cross sites, soils associated with SAT and TLA at the toe-slope positions showed significantly higher SWC, EC, P, K, and Cu contents than soils associated with PBA and SDI located at upper-slope positions (Supplementary Tables S1, S2). Significant Site × Plant species interactions were observed for soil pH, Ca, S, Fe, Al and Cr (*p* < 0.05; Tables 2, 3). Within the AMD-impacted site, these variables followed a pronounced topographic gradient. Specifically, toe-slope soils supporting SAT and TLA were characterized by acidic pH, low Ca contents, and elevated S, Fe and Al contents. In contrast, soils associated with SDI and PBA at mid- and upper-slope positions showed higher and more variable pH and Ca contents, with moderate S and Fe levels and low Al contents (Tables 2, 3). At the non-AMD-impacted site, these variables showed comparatively limited variation among plant species.

**Table 2:** Selected soil characteristics associated with four plant species at AMD-impacted and non-AMD-impacted sites, compared with Quebec soil background levels.

Soil parameter	Statistical effect ^2^	AMD-impacted site				Non-AMD-impacted site				Soil^1^ background levels for
		PBA^3^	SDI	SAT	TLA	PBA	SDI	SAT	TLA	Quebec (Canada) (mg/kg)
Soil Water Content (%)	P	13.8^4^	18.2	131.8	173.8	30.2	22.9	56.2	81.2	n.d.
pH	S × P	6.7a^5^	5.8a	3.3c	3.8c	5.5ab	5.6ab	5.4ab	5.6ab	n.d.
Electrical conductivity (μS/cm)	S, P	213.8	128.8	676.1	645.7	70.8	75.9	77.6	195.0	n.d.
Phosphorus (P) mg kg^−1^	P	457.9	448.0	637.6	626.5a	399.9	400.0	607.3	1207.4	n.d.
Potassium (K) mg kg^−1^	S, P	13742.3	16538.6	7618.0	6104.0	20504.3	19676.0	14318.0	14525.7	n.d.
Calcium (Ca) mg kg^−1^	S × P	35481.3a	17378.0ab	8511.4b	6309.6b	14791.1ab	16218.1ab	16595.9ab	16982.4ab	n.d.
Magnesium (Mg) mg kg^−1^	NS	9400.0	8969.3	5650.0	6116.7	7360.0	6766.7	7433.3	5766.7	n.d.
Sulfur (S) mg kg^−1^	S × P	1445.4bc	1148.1bc	13803.8a	6025.6ab	173.8 cd	85.1d	275.4 cd	955.0bc	n.d.
Copper (Cu) mg kg^−1^	S, P	182.0	112.2	182.0	182.0	20.0	19.5	35.5	53.7	65 (A), 100 (B), 500 (C)
Zinc (Zn) mg kg^−1^	NS	182.0	85.1	199.5	186.2	104.7	89.1	112.2	147.9	150 (A), 500 (B), 1,500 (C)
Iron (Fe) mg kg^−1^	S × P	50118.7ab	57544.0ab	213796.2a	177827.9ab	38018.9b	38459.2b	45383.7b	43112.8b	n.d.
Aluminium (Al) mg kg^−1^	S × P	16183.3ab	33800.0a	41184.3b	13316.7b	46900.0a	48566.7a	48633.3a	41500.0a	n.d
Molybdenum (Mo) mg kg^−1^	S	5.7	5.9	4.8	3.9	2.6	3.0	3.0	3.0	8 (A), 10 (B), 40 (C)

^1^Quebec soil criteria, according to Beaulieu (2021): A, background levels for the Superior Geological Province; n.d., not determined. ^2^Statistical effect S, significant site effect (*p* < 0.05); P, significant plant effect (*p* < 0.05); S × P = significant interaction (*p* < 0.05). NS, Not significant. ^3^Plant codes: PBA, *Populus balsamifera* (top-slope); SDI, *Salix discolor* (middle slope); SAT, *Scirpus atrocinctus* (toe-slope); TLA, *Typha latifolia* (toe-slope). ^4^Means are based on 2–6 samples (see Table 1). ^5^Scheffé’s post hoc tests were applied when the Site × Plant species interaction was significant. Within each row, values followed by the same letter do not differ significantly (Scheffé’s test, *p* < 0.05). In the absence of a significant interaction, Scheffé tests were applied to significant main effects only, and corresponding letter groupings are reported in the Supplementary Table S1.

**Table 3:** Selected metals and metalloids in soils associated with four plant species at AMD-impacted and non-AMD-impacted sites, compared to Quebec soil background levels (A), and regulatory limits (B, C).

Soil Parameter (mg kg^−1^)	Statistical effect^2^	AMD-impacted-site				Non-AMD-impacted site				Quebec (Canada) Soil^1^ Criteria	
		Soil associated with plant								Background levels (A)	Regulatory limits (B and C)
		PBA^3^	SDI	SAT	TLA	PBA	SDI	SAT	TLA	mg/kg	mg/kg
Arsenic (As)	NS	6.76^4^	5.13	9.33	6.60	4.47	3.89	6.31	7.41	5	B: 30 C: 50
Cobalt (Co)	NS	58.67	44.31	116.83	84.50	65.33	106.33	81.00	43.00	30	B: 50 C: 300
Chromium (Cr)	S × P	50.58a^5^	71.45a	32.66a	32.55a	69.29a	70.96a	99.54a	120.92a	100	B: 250 C: 800
Nickel (Ni)	NS	39.81	38.02	35.48	24.55	25.12	21.83	45.29	29.58	65	B: 1000 C: 500
Lead (Pb)	S	8.51	6.46	6.61	6.03	13.18	7.41	11.22	22.91	40	B: 5000 C: 1000
Manganese (Mn)	S	518.80	543.25	636.79	597.03	1150.80	997.70	1044.24	1184.68	1,000	B: 1000 C: 2200

^1^Quebec (Canada) soil criteria, according to Beaulieu (2021): Soil background levels (A) for the Superior geological province, Regulatory limits B: threshold for contaminants above background levels, C: the threshold above which contaminants pose a significant risk and require remediation to protect human health and the environment, n.d, not determined, ^2^Statistical effect S, significant site effect (*p* < 0.05); P, significant plant effect (*p* < 0.05); S × P = significant interaction (*p* < 0.05). ^3^PBA, *Populus balsamifera* (top-slope); SDI, *Salix discolor* (middle slope), SAT, *Scirpus atrocinctus* (toe-slope); TLA, *Typha latifolia* (toe-slope). ^4^Means are of 2 to 6 samples (see Table 1). ^5^Scheffé’s post hoc tests were applied when the Site × Plant species interaction was significant. Within each row, values followed by the same letter do not differ significantly (Scheffé’s test, *p* < 0.05). For Pb and Mn, the interaction was not significant but means and letter groupings of significant Site (S) factor are reported in the Supplementary Table S2.

Root mineral contents of P, Fe and Mn differed significantly between sites (*p* < 0.05; Table 4). Across species, root P and Mn contents were significantly lower at the AMD-impacted site, whereas root Fe content was significantly higher (Supplementary Table S3). Root contents of P, Ca, Zn, Fe, Mo and Mn also differed significantly among plant species (*p* < 0.05; Table 4 and Supplementary Table S3). Across sites overall, PBA and SDI accumulated more P, Ca and Zn, whereas SAT and TLA accumulated more Fe and Mn (Table 4; Supplementary Table S3). Significant Site × Plant species interactions for root K, S and Al contents were driven mainly by shifts in TLA roots at the AMD-impacted site (*p* < 0.05; Table 4).

**Table 4:** Selected root element contents of *Populus balsamifera* (PBA), *Salix discolor* (SDI), *Scirpus atrocinctus* (SAT), and *Typha latifolia* (TLA) sampled at an AMD-impacted site and a non-AMD-impacted site.

Root element content (mg kg^−1^)	Statistical effect^1^	AMD-impacted site				Non-AMD-impacted site			
		PBA	SDI	SAT	TLA	PBA	SDI	SAT	TLA
Phosphorus (P)	S, P	0.10^2^	0.07	0.03	0.04	0.15	0.15	0.08	0.07
Potassium (K)	S × P	0.47ab^3^	0.15b	0.39ab	1.22a	0.54a	0.35ab	0.53ab	0.78a
Calcium (Ca)	P	0.86	0.88	0.11	0.32	0.80	0.84	0.11	0.54
Sulfur (S)	S × P	0.02 cd	0.03 cd	0.40ab	0.69a	0.02 cd	0.02d	0.07bcd	0.11bc
Copper (Cu)	NS	37.12	56.13	25.62	36.64	9.00	9.00	14.62	10.87
Zinc (Zn)	P	141.74	148.22	38.20	57.61	135.61	89.83	35.76	35.34
Iron (Fe)	S, P	1851.40	2759.9	18285.21	41181.30	1256.03	1577.61	66645.00	13768.92
Aluminum (Al)	S × P	2323.3ab	1786.5b	1838.3b	2698.3ab	2380.0ab	3713.3ab	2399.5ab	11646.7a
Molybdenum (Mo)	P	4.37	4.34	4.00	3.72	3.86	4.53	3.68	3.72
Manganese (Mn)	S, P	31.61	66.30	257.16	230.57	87.34	92.79	1118.66	914.32

^1^Statistical effect S, significant site effect (*p* < 0.05); P, significant plant species effect (*p* < 0.05); S × P, significant site × plant species interaction (*p* < 0.05); NS, Not significant. ^2^Means are of 2 to 6 samples (see Table 1). ^3^Scheffé’s test was applied to root K, S, and Al contents since the Site × Plant species interactions were observed. Within each row, values followed by the same letter do not differ significantly at *p* < 0.05. In the absence of a significant interaction, Scheffé tests were applied to significant main effects only, and corresponding letter groupings are reported in the Supplementary Table S3.

Shoot Fe, Mo and Mn contents differed significantly between sites (*p* < 0.05), whereas shoot K, S, Fe, Al and Mn differed significantly among plant species (*p* < 0.05; Table 5). Across species, shoot Fe content was higher at the AMD-impacted site, whereas shoot Mo and Mn contents were lower (Supplementary Table S4). Across sites, TLA exhibited the highest shoot K content; SAT and TLA showed high shoot S, Fe and Mn contents, while PBA and SDI had comparatively low shoot contents (Supplementary Table S4). Significant Site × Plant species interactions were observed for shoot P, Ca, and Zn contents (*p* < 0.05; Table 5), with pronounced differences for PBA and SDI compared to SAT and TLA.

**Table 5:** Selected shoot element contents of *Populus balsamifera* (PBA), *Salix discolor* (SDI), *Scirpus atrocinctus* (SAT), and *Typha latifolia* (TLA) sampled at an AMD-impacted site and a non-AMD-impacted site.

Shoot element content (mg kg^−1^)	Statistical effect^1^	AMD-impacted site				Non-AMD-impacted site			
		PBA	SDI	SAT	TLA	PBA	SDI	SAT	TLA
Phosphorus (P)	S × P	0.12^2^ a^3^	0.08a	0.03b	0.02b	0.07ab	0.10a	0.10a	0.03ab
Potassium (K)	P	0.38	0.06	0.46	2.28	0.65	0.06	0.39	1.54
Calcium (Ca)	S × P	1.39a	1.33a	0.11c	0.45ab	0.35abc	0.95ab	0.24bc	0.92ab
Sulfur (S)	P	0.14	0.03	0.20	0.12	0.05	0.02	0.21	0.10
Copper (Cu)	NS	12.31	21.12	12.22	9.00	11.33	9.68	15.91	9.00
Zinc (Zn)	S × P	432.37a	283.67a	30.72c	23.92c	141.05ab	166.72a	40.19bc	17.12c
Iron (Fe)	S, P	656.14	778.04	5173.69	2305.68	399.67	286.22	1768.07	715.98
Aluminum (Al)	NS	1799.0	1664.5	1700.0	1700.0	1700.0	1700.0	3303.3	1893.3
Molybdenum (Mo)	S	3.82	4.50	4.59	4.03	4.83	4.91	6.49	5.06
Manganese (Mn)	S, P	20.61a	37.44a	336.98a	367.62a	81.08a	46.76a	1875.86a	2584.04a

^1^Statistical effect S, significant site effect (*p* < 0.05); P, significant plant species effect (*p* < 0.05); S × P, significant site × plant species interaction (*p* < 0.05). ^2^Means are of 2 to 6 samples (see Table 1). ^3^Scheffé’s post hoc tests were applied when the Site × Plant species interaction was significant. Within each row, values followed by the same letter do not differ significantly (Scheffé’s test, *p* < 0.05). In the absence of a significant interaction, Scheffé tests were applied to significant main effects only, and corresponding letter groupings are reported in the Supplementary Table S4.

### 3.2 Bioconcentration (BCF) and translocation factors (TF)

Root bioconcentration factors (RBCF) differed significantly between sites for P and Mo, with lower values observed at the AMD-impacted site compared with the non-AMD-impacted site (*p* < 0.05; Table 6; Supplementary Table S5A). RBCF also differed significantly among plant species for P, Ca, Zn, Fe, Al and Mn (*p* < 0.05; Table 6). Across sites, RBCF values were higher in PBA and SDI for P, Ca, and Zn, whereas SAT and TLA showed higher RBCF for Fe, Al and Mn (Table 6). Significant Site × Plant species interactions were observed for K-RBCF and S-RBCF (*p* < 0.05; Table 6). K-RBCF was highest for TLA at the AMD-impacted site, whereas PBA and SDI showed lower values. For S-RBCF, SAT and SDI at the non-AMD-impacted site showed the highest values, while values at the AMD-impacted site were more variable.

**Table 6:** Average root and shoot bioconcentration factors (RBCF and SBCF) and translocation factor (TF) of *Populus balsamifera* (PBA), *Salix discolor* (SDI), *Scirpus atrocinctus* (SAT) and *Typha latifolia* (TLA) across an AMD-impacted site and a non-AMD-impacted site.

Element	Statistical effect^1^			Root BCF (RBCF)				Shoot BCF (SBCF)				Translocation factor (TF)			
	RBCF	SBCF	TF	PBA^2^	SDI	SAT	TLA	PBA	SDI	SAT	TLA	PBA	SDI	SAT	TLA
Phosphorus (P)	S, P	P	NS	3.6 10^–5/3^A^/4^	5.1 10^−5^A	9.8 10^−6^B	3.4 10^−5^B	2.16 A	2.02 A	0.80 AB	0.33 B	0.93	1.13	1.26	0.73
Potassium (K)	S × P	P	P	3.0 10^−5^	1.3 10^−5^	4.0 10^−5^	1.1 10^−4^	3.0 10^−5^B	3.6 10^−6^C	6.4 10^−5^AB	1.6 10^−4^A	1.06AB	0.21 B	1.41 A	1.57A
Calcium (Ca)	P	P	NS	3.6 10^−5^A	5.1 10^−5^A	9.8 10^−6^B	3.4 10^−5^A	3.0 10^−5^AB	6.7 10^−5^A	1.4 10^−5^ B	6.1 10^−5^A	1.08	1.37	1.40	1.94
Sulfur (S)	S × P	S	P	4.3 10^−5^	7.5 10^−5^	8.9 10^−5^	1.2 10^−4^	1.7 10^−4^	7.3 10^−5^	1.1 10^−4^	4.9 10^−5^	4.00 A	0.98AB	1.31AB	0.42B
Copper (Cu)	NS	SxP	NS	0.30	0.48	0.24	0.20	0.20 AB	0.31 A	0.18 AB	0.09 B	0.90	0.79	0.96	0.58
Zinc (Zn)	P	P	SxP	1.01 AB	1.32 A	0.26 B	0.27 B	1.80 A	2.49 A	0.23 B	0.12 B	2.18	1.91	0.99	0.57
Iron (Fe)	P	NS	P	0.03 B	0.04 B	0.11 AB	0.27 A	1.1 10^−2^	1.0 10^−2^	3.1 10^−2^	1.5 10^−2^	0.34 A	0.23AB	0.20AB	0.05B
Aluminum (Al)	P	S, P	S	0.06 B	0.06 B	0.09 AB	0.19 A	0.04 AB	0.04 B	0.08 A	0.08 A	0.80	0.76	0.80	0.63
Manganese (Mn)	P	S, P	P	0.07 B	0.11 B	0.65 A	0.55 A	0.07 B	0.11 B	0.65 A	0.55 A	0.78AB	0.53 B	1.65AB	2.12A
Molybdenum (Mo)	S	S, P	S, P	1.10	1.05	1.0	1.1	1.16	1.12	1.44	1.32	1.05	1.06	1.63	1.22

^1^Statistical effect S, significant site effect (*p* < 0.05); P, significant plant species effect (*p* < 0.05); S × P, significant site × plant species interaction (*p* < 0.05). ^2^PBA, *Populus balsamifera* (top-slope); SDI, *Salix discolor* (middle slope), SAT, *Scirpus atrocinctus* (toe-slope); TLA, *Typha latifolia* (toe-slope). ^3^Means are of 2 to 6 samples (see Table 1). ^4^Scheffé’s post hoc tests were applied when the Site × Plant species interaction was significant. Within each row, values followed by the same letter do not differ significantly (Scheffé’s test, *p* < 0.05). Results for the main effect of Site are presented in Supplementary Table S5A.

Shoot bioconcentration factors (SBCF) differed significantly between sites for S, Cu, Al, Mn and Mo (*p* < 0.05; Table 6; Supplementary Table S5A). Across plant species, SBCF values for S, Cu, and Mo were significantly lower at the AMD-impacted site, whereas SBCF for Al and Mn were higher. SBCF also differed significantly among plant species for P, K, Ca, Cu, Zn, Fe, Al and Mn (*p* < 0.05; Table 6). Across sites, SBCF values for these elements were generally higher in PBA and SDI and lower in SAT and TLA.

Translocation factors (TF) differed significantly among elements, sites, and plant species (*p* < 0.05; Table 6; Supplementary Table S5A). At the site level, Zn and Mo generally exhibited TF values close to or greater than 1, whereas Cu and Fe consistently showed TF values lower than 1, indicating contrasting patterns of element partitioning between roots and shoots. TF values also differed among plant species, with higher values for Zn and Mo in PBA and SDI compared to SAT and TLA, while Cu and Fe remained poorly translocated across all species. Together, these results highlight element- and plant-specific differences in internal element partitioning along the soil-root-shoot continuum.

### 3.3 Estimation of fungal abundance

**Figure 2:**
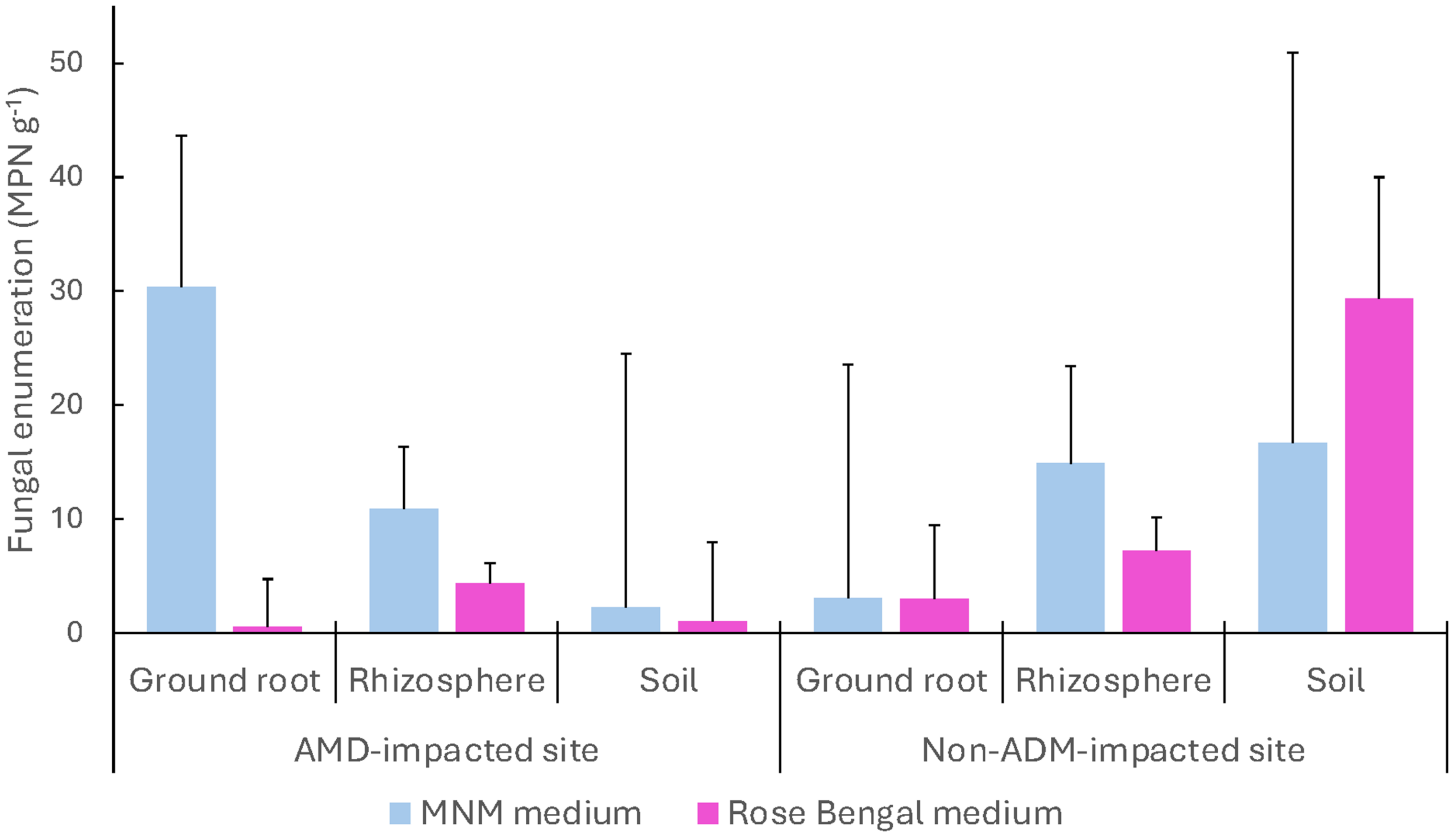
Culturable fungal estimates across root zones, sites, and culture media. The Most Probable Number (MPN) method was used to estimate fungal abundance and was expressed as MPN g^−1^ dry matter. Although the ANOVA indicated a significant effect (*p* < 0.05), no significant pairwise differences among treatments were detected using Scheffé’s post hoc test.

Culturable fungal estimates were significantly affected by the three-way interaction among Site, Root zone, and Culture medium (*p* < 0.05; Figure 2). To provide an overall description of fungal estimates, descriptive means are reported for each factor independently. When averaged across plant species, root zones and culture media, mean culturable fungal estimates were lower at the AMD-impacted site (8.5 MPN g^−1^ dry matter[dm]) than at the non-AMD-impacted site (9.5 MPN g^−1^ dm). Across plant species, mean fungal estimates were highest in SDI (16.4 MPN g^−1^ dm), followed by PBA (11.1 MPN g^−1^ dm), SAT (5.6 MPN g^−1^ dm), and TLA (2.6 MPN g^−1^ dm). Across root zones, fungal estimates were highest in the bulk soil (9.7 MPN g^−1^ dm), followed by the rhizosphere (9.3 MPN g^−1^ dm) and roots (7.6 MPN g^−1^ dm). Across culture media, higher fungal estimates were observed on MMN medium (11.5 MPN g^−1^ dm) than on RB medium (6.2 MPN g^−1^ dm).

### 3.4 Root fungal identification

To maximize taxonomic coverage, only one distinct fungal phenotype was retained per plant and per root sample. A total of 126 isolates were obtained from MMN cultures, including 64 isolates from surface-disinfected root pieces and 62 from dilution-based MPN estimation of ground-root homogenates. Among these, 96 isolates were successfully sequenced and identified at least to the order level. The identified isolates were dominated by *Ascomycota* (73%), followed by *Basidiomycota* (18%) and *Mucoromycota* (9%).

**Figure 3:**
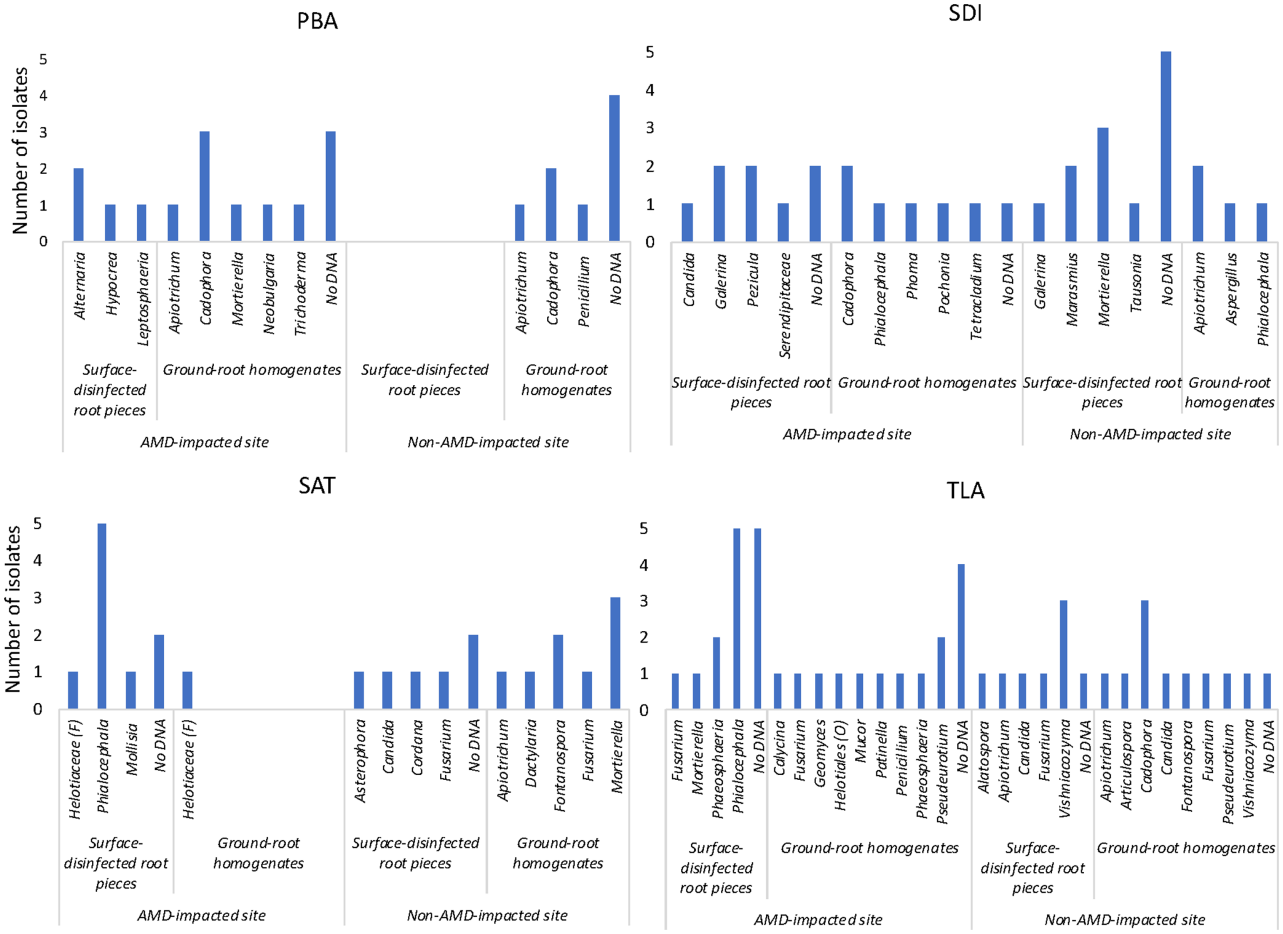
Diversity and distribution of cultivable root-associated fungi isolated from *Populus balsamifera* (PBA), *Salix discolor* (SDI), *Scirpus atrocinctus* (SAT), and *Typha latifolia* (TLA) at AMD-impacted and non-AMD-impacted sites. Bars represent the number of isolates per taxon from root surface-disinfected root pieces and ground-root homogenates. “No DNA” indicates isolates for which molecular identification was unsuccessful. Taxa indicated at the family (F) or order (O) level correspond to isolates for which reliable genus-level identification was not achieved.

For fungal isolates from the surface-disinfected root pieces, 13 genera were exclusive to the AMD-impacted site, 11 were exclusive to the non-AMD-impacted site, and 4 were shared between sites (Figure 3), whereas for ground-root homogenates, 17 genera were exclusive to the AMD-impacted site, 14 were identified to genus, family, or order level at the non-AMD-impacted site, and 6 genera were common to both sites.

Within Ascomycota, *Phialocephala* spp. were isolated from SDI, SAT, and TLA roots at the AMD-impacted site. *Cadophora* spp. were recovered mainly from ground-root homogenates of PBA at both sites, with *C. malorum* isolated from SDI and *C. luteo-olivacea* from TLA roots at both sites. *Mortierella* spp. were isolated from all plant species: *M. alpina* was recovered from PBA roots at the AMD-impacted site, whereas *Mortierella* spp. and *M. echinula* were isolated from SDI roots at the non-AMD-impacted site. From SAT ground-root homogenates, *Mortierella* sp. and *M. elongata* were isolated at the non-AMD-impacted site, while *M. fimbricystis* was isolated from TLA roots at the AMD-impacted site. *Phaeosphaeria spartinicola* was isolated exclusively from TLA roots at the AMD-impacted site. Additional isolates included *Apiotrichum porosum* and *Mortierella* spp., primarily from the non-AMD-impacted site. *Fusarium acuminatum* was isolated from TLA roots at both sites. Most *Basidiomycota*, including *Galerina* sp. and members of *Serendipitaceae*, were isolated from SDI at both sites. Yeast species included *Apiotrichum porosum* (*Trichosporonaceae*), *Candida* spp. (*Saccharomycetaceae*) and *Pseudeurotium bakeri* (*Pseudeurotiaceae*). *Apiotrichum porosum* and *Candida* spp. were predominantly isolated from ground-root homogenates of all plants at the non-AMD-impacted site, whereas *Pseudeurotium bakeri* was isolated from TLA roots at both sites.

### 3.5 Relationship between mineral content and root-associated fungi

**Figure 4:**
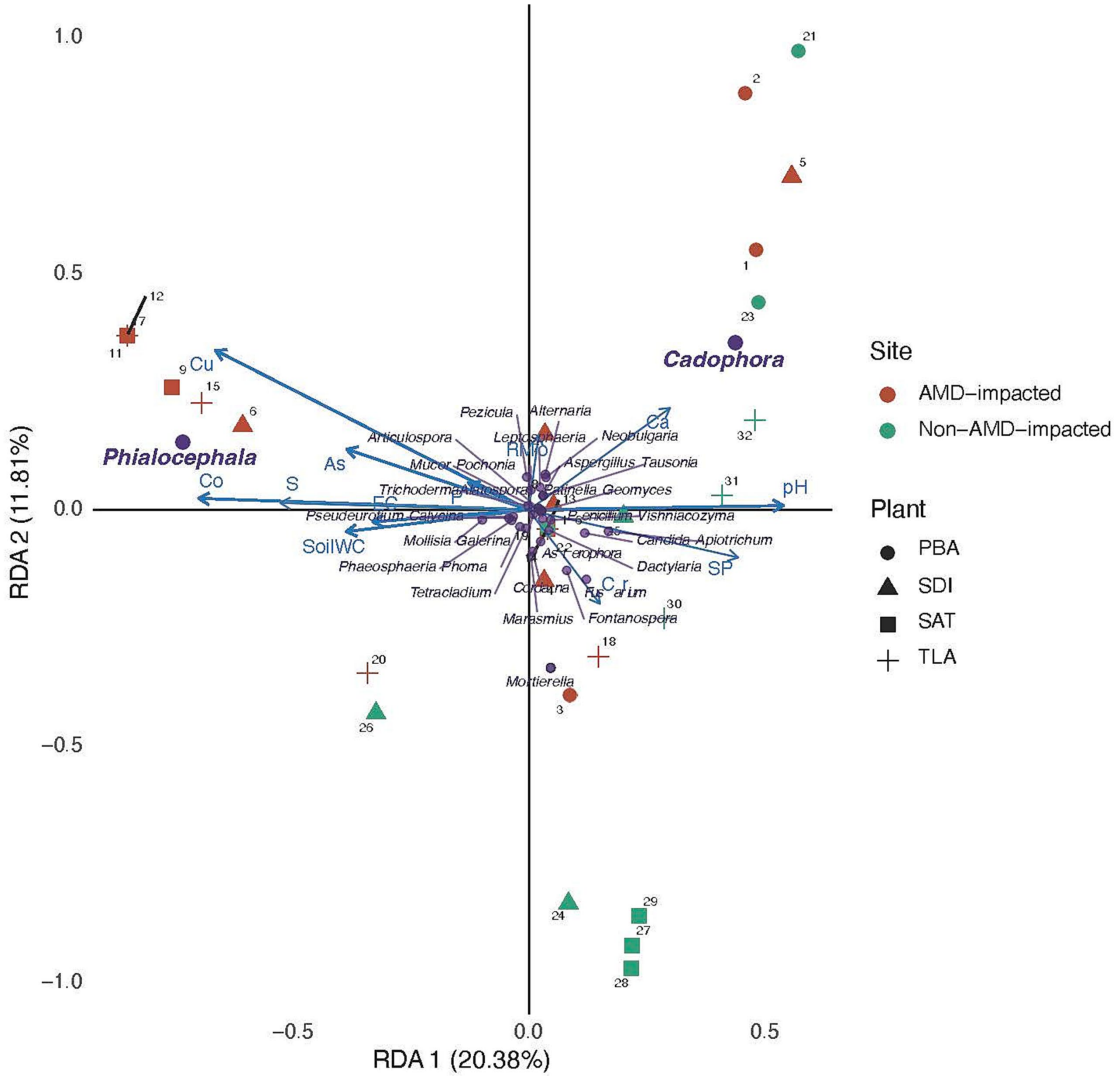
Triplot of the redundancy analysis (RDA) relating soil and plant mineral parameters to fungal genera at AMD-impacted (orange) and non-AMD-impacted (green) sites. Each purple point represents a fungal genus, and each colored point represents a sample, with symbols indicating the host plant. Arrow length and direction indicate the strength and orientation of associations with the ordination axes, and angles between arrows reflect relationships among variables. Element abbreviations follow standard chemical notation (e.g., Cu = copper), and R- and S- denote root and shoot element contents, respectively. Bioconcentration factors (BCF) and element translocation factors (TF) are indicated as element + plant compartment (R or S) + BCF (e.g., CuSBCF). The RDA model was significant based on permutation tests (*p* < 0.05).

Redundancy analysis (RDA) revealed strong associations between soil physicochemical properties, plant mineral parameters, and root-associated fungal genera. The model explained 63% of the total variance (adjusted R^2^), indicating an acceptable fit after accounting for model complexity (Supplementary Table S6). The first two canonical axes accounted for 20.4% (RDA1) and 11.8% (RDA2) of the total variance, respectively (Figure 4). Permutation tests identified highly significant effects for Cu, KTF, Ca, P, SP, SMo, SMn, Co, KRBCF, ZnTF, CuTF, MnTF, Cr, ZnSBCF, PSBCF, RMo, SZn, AlTF, S, EC and SoilWC, AlSBCF and As (Supplementary Table S7).

At the AMD-impacted site, *Phialocephala* spp. were associated mainly with RDA1 and aligned with higher soil Cu, Co, S, and As contents, as well as increased SWC and EC. *Phialocephala* spp. also showed positive associations with MnTF and AlSBCF, and negative associations with PSBCF and Cr. These variables were elevated at the AMD-impacted site, particularly in association with SAT and TLA.

*Cadophora* spp. were mainly associated with RDA2, showing weak positive associations with soil Ca content, KTF, SZn and RMo, and negative associations with soil P content and RK. These parameters were most prominent in association with PBA at both sites.

At the non-AMD-impacted site, *Mortierella* spp. marginally correlated positively with RDA1 and negative with RDA2, aligning with plant-related variables such as Ca, KTF, and Zn-TF. Overall, these parameters were particularly associated with SDI and PBA. Several other fungal taxa were located near the origin of the ordination space, indicating weak or no association with the measured explanatory variables.

## 4 Discussion

### 4.1 Soil and plant characteristics at AMD and non-AMD-impacted sites

The AMD-impacted site exhibited pronounced topographic and hydrological contrasts, with acidic and metal-rich soils occurring in water-saturated toe-slope positions where SAT and TLA were established, while mid- and upper-slope soils supporting SDI and PBA were characterized by higher pH values, reflecting less extreme chemical conditions. Overall, the AMD-impacted site was characterized by higher SWC and EC, along with elevated concentrations of metals and sulfur, indicative of ongoing sulfide oxidation. The concentrations of several metals, including Cu, Zn, As, and Co, exceeded Quebec background levels (Beaulieu, 2021), although values were lower than those reported prior to site restoration (Lamont Inc, 2017), suggesting partial attenuation following remediation efforts.

The spatial variability in soil pH observed across the AMD-impacted site likely reflects past remediation efforts involving neutralizing amendments, which primarily influenced well-drained mid- and upper-slope positions rather than poorly drained areas. In these zones, amendment incorporation was effective and resulted in moderately higher soil pH values. In contrast, water-saturated toe-slope soils remained highly acidic and metal-rich, likely reflecting continued sulfide oxidation. As a result, SAT and TLA grew under the most chemically stressful conditions, characterized by low pH, high SWC, elevated EC, and high sulfur contents.

By comparison, the non-AMD-impacted site was characterized by moderately acidic to sub-neutral pH (5.4–5.6), with limited variation among plant species and no pronounced topographic gradient. Differences in soil acidity and water saturation between sites influenced nutrient and metal contents in plant roots and shoots. Soil pH constrains the mobility of many elements while enhancing that of others (McCauley et al., 2017), and sulfur oxidation contributes to increased EC and metal solubility (Rather et al., 2022). Consistent with these processes, patterns of element accumulation and translocation varied with site conditions and host species. Similar responses have been reported for *Populus* and *Salix* species, as well as helophytic plants such as *Scirpus* and *Typha,* growing in metal-impacted environments (Brunham and Bendell, 2011; Cao et al., 2020; Tőzsér et al., 2023; Wani et al., 2020).

The gradients of acidity, EC, metal availability, and SWC observed along the hill slope at the AMD-impacted site likely act as ecological filters shaping soil microbial communities, in contrast to the more homogeneous conditions at the non-AMD-impacted site. Previous studies indicate that metal-contaminated environments tend to promote plant–fungal associations (Ważny et al., 2023) and define the abiotic context within which these interactions occur.

### 4.2 Selective enrichment of root-associated fungi

Differences in culturable fungal estimates across root compartments and culture media revealed clear patterns across functional groups. At the non-AMD-impacted site, fungal estimates on Rose Bengal medium were highest in bulk soil and declined toward roots, in agreement with the dominance of free-living saprotrophic fungi under comparatively low environmental stress. In contrast, at the AMD-impacted site, fungal estimates on MMN medium were highest in root compartments and declined from roots to bulk soil, indicating selective enrichment of fungi associated with plant roots under AMD-related conditions.

Importantly, higher MPN values reflect relative enrichment on selective media rather than absolute fungal abundance. Comparable spatial enrichment of root-associated fungi along pH and metal gradients has been reported for *Arabidopsis arenosa* growing on metal-rich soils and in the rhizosphere of *Phragmites australis* at AMD-impacted sites characterized by acidic pH and elevated Fe, Cu, and Zn contents (Kalu et al., 2021). More broadly, soil characteristics and host plant identity are known to structure microbial communities in the root zone (Cao et al., 2020; Kalu et al., 2021; Ważny et al., 2023), supporting the view that AMD-related edaphic stress acts as a selective filter favoring root-associated fungi over free-living saprotrophic taxa.

### 4.3 Root fungal dominance under stress

Fungal taxa isolated from the AMD-impacted site closely resembled communities previously reported from mining wastes, acid mine drainage streams, and contaminated soils worldwide (Gagnon et al., 2020; Kalu et al., 2021). Several of these taxa have been widely documented as stress-tolerant saprotrophs or endophytes adapted to acidic, metal-rich, and water-saturated environments (Behera and Das, 2023; Liao et al., 2019; Ruotsalainen et al., 2022). A substantial proportion of isolates displayed melanized hyphae, a morphological trait commonly associated with tolerance to multiple abiotic stressors (Berthelot et al., 2019; Jumpponen and Trappe, 1998; Malicka et al., 2022).

Among these fungi, dark septate endophytes (DSE) represent a distinct root-associated ecological and functional group characterized by stress adaption and broad ecological plasticity. DSE comprise a diverse assemblage of saprotrophs, opportunistic pathogens, and symbiotic fungi, many of which are likely facultative mutualists in disturbed or contaminated environments. Their ecological relevance is broad and includes metal sequestration, melanin-mediated binding of toxic elements and the modulation of plant mineral nutrition and stress tolerance (Domka et al., 2019; Jumpponen and Trappe, 1998). Together, these characteristics position DSE as key components of stress-adapted root fungal assemblages in chemically stressed environments.

Within this DSE assemblage, *Phialocephala* spp. and *Cadophora* spp. were particularly prominent and displayed contrasting ecological associations. *Phialocephala* spp. were mainly associated with SAT and TLA in water-saturated toe-slope positions at the AMD-impacted site, in line with adaptation to acidic and metal-rich conditions. *Phialocephala bamuru* was frequently isolated from surface-disinfected root pieces of both plant species. In contrast, *Cadophora* spp. were more commonly associated with mid- to upper-slope positions characterized by drier, moderately acidic soils. In these zones, *Phialocephala europaea, Cadophora malorum*, and *C. luteo-olivacea* were primarily recovered from ground-root homogenates. Differences in fungal recovery between isolation approaches likely reflect methodological influences rather than clear differences in fungal localization within root tissues (Barrow, 2003; Jumpponen and Trappe, 1998; Malicka et al., 2022).

Beyond the DSE assemblage, genera such as *Phaeosphaeria* and *Fusarium* were recovered from both AMD-impacted and non-AMD-impacted sites. These fungi are often regarded as generalist saprotrophs or opportunistic pathogens, but under environmental stress conditions, they may also act as facultative root colonizers. Conversely, fungal assemblages from the non-AMD-impacted site included a higher proportion of generalist saprotrophs such as *Penicillium* and *Aspergillus* (Malicka et al., 2022), as well as decomposers and aquatic-associated taxa such as *Articulospora* and *Fontanospora* (Behera and Das, 2023). Furthermore, yeast generalists involved in carbon cycling, including *Apiotrichum* and *Vishniacozyma*, were also frequently isolated. Similarly, *Mortierella* spp., widely recognized as generalist saprotrophs or root-associated fungi, were recovered from multiple plant species and sites. Mycorrhizal fungi, i.e., root symbiotic fungi forming nutrient-exchange associations with host plants, were detected only in *Salix discolor* roots and one Serendipitaceae isolate. No additional mycorrhizal fungi were detected in other plant host species, despite their potential to form mycorrhizal associations (Davison et al., 2021), likely reflecting methodological and environmental constraints rather than true absence.

Local field and soil characteristics influenced fungal recovery patterns, highlighting the interpretative limits of culture-based approaches. For PBA at the non-AMD-impacted site, the lack of isolates from surface-disinfected root pieces likely reflects sampling constraints rather than fungal absence. Although fine roots were visible, excavation in dry clay-loam soils at upper-slope positions frequently resulted in breakage of deeper roots, favoring fungal recovery from ground-root homogenates. Despite their inherent selective bias, culture-based approaches enable the isolation of stress-tolerant and functionally relevant fungal taxa that may play key roles in supporting plant performance in AMD-impacted environments (James et al., 2020).

### 4.4 Relationship between soil–plant mineral parameters and identified root-associated fungi

By integrating fungal occurrence with soil and plant mineral parameters, this analysis extends beyond species-level patterns to examine how root-associated fungi are structured along soil–plant mineral gradients. Root-associated fungi have been shown to influence plant mineral nutrition and persistence along gradients of soil acidity and metal availability in AMD-impacted ecosystems (Muthusaravanan et al., 2018; Shi et al., 2023; Yaashikaa et al., 2022). Redundancy analysis revealed clear associations of *Phialocephala* spp. and *Cadophora* spp. with multiple soil and plant mineral parameters, reflecting strong environmental influence on soil chemistry and plant mineral status across sites.

*Phialocephala* spp. were closely associated with water-saturated and metal-rich soils, where shifts in plant mineral allocation were also observed. Consistent with these associations, tolerance of *Phialocephala* spp. to elevated metal concentrations has been documented previously, including for *P. malorum* (Malicka et al., 2022). In acidic, metal-rich environments, the recurrent association of *Phialocephala* spp. with altered soil and plant mineral profiles suggests that this genus belongs to a stress-adapted group of root-associated fungi that may contribute to plant establishment under edaphic stress.

The associations observed for *Phialocephala* spp. in the RDA, particularly under arsenic-rich and acidic conditions, are consistent with altered phosphorus acquisition, in line with known interactions between phosphate and arsenate uptake pathways (Kabata-Pendias, 2011). Similarly, Haruma et al. (2021) showed that *Miscanthus sinensis* inoculated with *Phialocephala fortini* exhibited reduced variability in shoot and root mineral contents when grown in mine soils, suggesting that *Phialocephala* spp. may influence mineral mobility and plant nutrition under acidic soils.

Patterns observed for *Cadophora* indicate an association with plant micronutrient regulation, particularly zinc, under moderately acidic to near-neutral soils. These results align with previous findings reporting enhanced Zn nutrition in *Salix caprea* associated with *Cadophora finlandica* (Berthelot et al., 2019). The occurrence of *Cadophora* across multiple hosts and sites suggests a broad ecological range and a potential role as a beneficial root endophyte.

Taken together, these findings indicate that *Phialocephala* spp. and *Cadophora* spp. occupy distinct ecological positions along AMD-driven soil–plant mineral gradients and represent functionally relevant components of root-associated fungal assemblages in stressed environments.

## 5 Conclusion

This study examined soil physicochemical properties, plant mineral contents, and culturable root-associated fungi under contrasting topographic and hydrological conditions at an AMD-impacted site and a nearby non-impacted reference site. Pronounced gradients in soil acidity, water saturation, and metal availability were evident along the soil-root-shoot continuum, with the water-saturated toe-slope zones emerging as the most chemically stressed. Together, these findings link soil and plant mineral gradients to patterns of culturable root-associated fungal composition across AMD-influenced environmental gradients.

Culturable fungal abundance and taxonomic composition varied markedly between sites and root compartments. At the non-AMD-impacted site, fungal assemblages were dominated by generalist saprotrophic species typical of less chemically constrained soils, whereas acidic, metal-rich, and water-saturated conditions at the AMD-impacted site selectively favored stress-adapted root-associated fungi, particularly dark septate endophytes (DSE). These patterns highlight the strong selective influence of edaphic conditions on culturable root-associated fungal assemblages.

Within the DSE assemblage, *Phialocephala* spp. were consistently associated with plant hosts established under the most extreme physicochemical conditions at the AMD-impacted site, suggesting trait-based filtering under combined gradients of acidity, hydrology, and metal availability. In contrast, *Cadophora* spp. exhibited broader host and site associations under moderately acidic conditions, in agreement with greater ecological plasticity. Although these associations point to potential functional relevance, further studies under controlled conditions will be required to confirm the roles of these fungal species in metal sequestration and plant mineral nutrition.

Overall, this study demonstrates that culturable root-associated fungi respond predictably to edaphic gradients and that distinct, stress-tolerant fungal groups are consistently associated with plant establishment in AMD-impacted environments. While culture-dependent approaches capture only a subset of total fungal diversity, they provide valuable access to stress-adapted species that may contribute to plant persistence under extreme conditions. These findings highlight that successful fungal-assisted phytoremediation of AMD-impacted soils requires strategies tailored to specific edaphic gradients, plant traits, and fungal-plant associations.

## Data Availability

The raw data supporting the conclusions of this article will be made available by the authors, without undue reservation.
