# Supplementary material for: Root-associated fungi in acid mine drainage-impacted environments
Source: Front Microbiol. 2026 Jun 10;17:1812818. doi: 10.3389/fmicb.2026.1812818 (PMC13293307; doi:10.3389/fmicb.2026.1812818)
Supplement: Supplementary file 1 [file table_1.docx]

**Supplementary Table S1. Main effects of site and plant species on soil physicochemical characteristics**

| Parameter | Unit | AMD-impacted site | Non-AMD-impacted site | **PBA** | **SDI** | **SAT** | **TLA** |
| --- | --- | --- | --- | --- | --- | --- | --- |
| Soil Water Content | % | **49.26** | **41.97** | **20.43 B** | **20.53 B** | **85.11 A** | **117.49 A** |
| **pH** | – | **4.90** | **5.51** | **6.09** | **5.68** | **4.36** | **4.71** |
| **Electrical conductivity** | µS cm⁻¹ | **330.14 a** | **94.82 b** | **122.80 B** | **98.13 B** | **230.78 AB** | **352.29 A** |
| Phosphorus (P) | mg kg⁻¹ | **535.06** | **585.19** | **427.96 B** | **423.35 B** | **622.30 AB** | **869.76 A** |
| Potassium (K) | mg kg⁻¹ | **11 001 b** | **17 256 a** | **17 123 AB** | **18 107 A** | **10 968 BC** | **10 315 C** |
| Calcium (Ca) | mg kg⁻¹ | **13 508.28** | **16 127.24** | **22 924.51** | **16 722.45** | **11 904.19** | **10 396.81** |
| Magnesium (Mg) | mg kg⁻¹ | **7 533.99** | **6 833.33** | **8 383.33** | **7 867.97** | **6 541.67** | **5 941.67** |
| Sulfur (S) | mg kg⁻¹ | **3 434.79** | **250.55** | **504.66** | **314.70** | **1 956.59** | **2 383.97** |
| Copper (Cu) | mg kg⁻¹ | **160.88 a** | **29.32 b** | **59.92 A** | **46.73 A** | **80.24 A** | **99.04 A** |
| Zinc (Zn) | mg kg⁻¹ | **154.88** | **111.46** | **137.06** | **87.30** | **149.83** | **166.30** |
| Iron (Fe) | mg kg⁻¹ | **102 352.86 a** | **41 171.81 b** | **43 591.32** | **47 304.23** | **98 469.11** | **87 458.09** |
| Aluminium (Al) | mg kg⁻¹ | **2150** | **4620** | **4000** | **4430** | **2500** | **2230** |
| Molybdenum (Mo) | mg kg⁻¹ | **5.38 A** | **2.92 B** | **4.17** | **4.50** | **4.33** | **3.58** |

No significant Site × Plant species interaction was detected for the parameters shown. Scheffé post hoc tests were applied to significant main effects only (*p <* 0.05). Within each row, different lower-case letters indicate significant differences between Sites, whereas different upper-case letters indicate significant differences among Plant species.
