# Supplementary material for: Root-associated fungi in acid mine drainage-impacted environments
Source: Front Microbiol. 2026 Jun 10;17:1812818. doi: 10.3389/fmicb.2026.1812818 (PMC13293307; doi:10.3389/fmicb.2026.1812818)
Supplement: Supplementary file 2 [file table_2.docx]

Supplementary Table S2. Main effects of site and plant species on metal and metalloid contents in soil.

| Soil parameter | Unit | AMD-impacted site | Non-AMD-impacted site | PBA | SDI | SAT | TLA |
| --- | --- | --- | --- | --- | --- | --- | --- |
| Arsenic (As) | mg kg⁻¹ | 6.80 | 5.37 | 5.47 | 4.51 | 7.68 | 7.05 |
| Cobalt (Co) | mg kg⁻¹ | 76.08 | 73.92 | 62.00 | 73.33 | 98.92 | 63.75 |
| Chromium (Cr) | mg kg⁻¹ | 44.29 | 87.74 | 59.21 | 71.20 | 57.07 | 62.75 |
| Nickel (Ni) | mg kg⁻¹ | 34.03 | 29.34 | 31.81 | 18.80 | 40.00 | 27.20 |
| Lead (Pb) | mg kg⁻¹ | 6.86 b | 12.62 a | 10.63 | 6.89 | 8.69 | 11.79 |
| Manganese (Mn) | mg kg⁻¹ | 572 b | 1092 a | 773 | 736 | 815 | 842 |

No significant Site × Plant species interaction was detected for the parameters shown. Scheffé post hoc tests were applied to significant main effects only (*p <* 0.05). Within each row, different lower-case letters indicate significant differences between Sites (Scheffé’s test, *p <* 0.05). No letters are shown for Plant species when the main effect was not significant.
