# Supplementary material for: Root-associated fungi in acid mine drainage-impacted environments
Source: Front Microbiol. 2026 Jun 10;17:1812818. doi: 10.3389/fmicb.2026.1812818 (PMC13293307; doi:10.3389/fmicb.2026.1812818)
Supplement: Supplementary file 3 [file table_3.docx]

### Supplementary Table S3. Main effects of site and plant species on **root element contents**

| **Root element** | **Unit** | **Site** | | **Plant species** | | | |
| --- | --- | --- | --- | --- | --- | --- | --- |
| **content** |  | **AMD-impacted** | **Non-AMD-impacted** | **PBA** | **SDI** | **SAT** | **TLA** |
| **Phosphorus (P)** | mg kg⁻¹ | 0.06 b | 0.11 a | 0.13 A | 0.11 AB | 0.04 AB | 0.05 B |
| **Potassium (K)** | mg kg⁻¹ | 0.47 | 0.55 | 0.51 | 0.21 | 0.43 | 1.03 |
| **Calcium (Ca)** | mg kg⁻¹ | 0.47 | 0.62 | 0.83 A | 0.86 A | 0.11 C | 0.39 B |
| **Sulfur (S)** | mg kg⁻¹ | 0.11 | 0.04 | 0.02 | 0.02 | 0.17 | 0.28 |
| **Copper (Cu)** | mg kg⁻¹ | 37.39 a | 10.65 b | 18.28 | 22.47 | 19.36 | 19.95 |
| **Zinc (Zn)** | mg kg⁻¹ | 82.45 | 62.63 | 138.64 A | 115.39 A | 36.96 B | 45.12 B |
| **Iron (Fe)** | mg kg⁻¹ | 7 876.08 a | 3 673.05 b | 1 525.05 B | 2 087.59 B | 11 039.47 A | 23 812.02 A |
| **Aluminum (Al)** | mg kg⁻¹ | 1996.64 | 3515.60 | 2235.63 | 2454.71 | 2175.70 | 4125.72 |
| **Molybdenum (Mo)** | mg kg⁻¹ | 4.11 | 3.95 | 4.12 AB | 4.44 A | 3.84 AB | 3.72 B |
| **Manganese (Mn)** | mg kg⁻¹ | 301.72 b | 105.58 a | 52.54 B | 78.43 B | 536.29 A | 459.20 A |

No significant Site × Plant species interaction was detected for the parameters shown. Scheffé post hoc tests were applied to significant main effects only (*p <* 0.05). Within each row, different lower-case letters indicate significant differences between Sites, whereas different upper-case letters indicate significant differences among Plant species.
