# Supplementary material for: Root-associated fungi in acid mine drainage-impacted environments
Source: Front Microbiol. 2026 Jun 10;17:1812818. doi: 10.3389/fmicb.2026.1812818 (PMC13293307; doi:10.3389/fmicb.2026.1812818)
Supplement: Supplementary file 4 [file table_4.docx]

Supplementary Table S4. Main effects of site and plant species on **shoot element contents**

| **Shoot element** |  | **Site** | | **Plant species** | | | |
| --- | --- | --- | --- | --- | --- | --- | --- |
| **content** | **Unit** | **AMD-impacted** | **Non-AMD-impacted** | **PBA** | **SDI** | **SAT** | **TLA** |
| **Phosphorus (P)** | mg kg⁻¹ | **0.05** | **0.07** | **0.09** | **0.09** | **0.05** | **0.03** |
| **Potassium (K)** | mg kg⁻¹ | **0.42** | **0.70** | **0.50 B** | **0.07 C** | **0.53 B** | **1.36 A** |
| **Calcium (Ca)** | mg kg⁻¹ | **0.46** | **0.52** | **0.70** | **1.17** | **0.14** | **0.57** |
| **Sulfur (S)** | mg kg⁻¹ | **0.17** | **0.19** | **0.09 A** | **0.02 B** | **0.21 A** | **0.12 A** |
| **Copper (Cu)** | mg kg⁻¹ | **12.75** | **11.49** | **11.82** | **15.52** | **13.43** | **9.00** |
| **Zinc (Zn)** | mg kg⁻¹ | **97.43** | **63.43** | **246.95** | **217.62** | **35.14** | **20.24** |
| **Iron (Fe)** | mg kg⁻¹ | **1 580.36 a** | **616.88 b** | **512.15 B** | **471.74 B** | **3024.13 A** | **1226.31 AB** |
| **Aluminum (Al)** | mg kg⁻¹ | **1696** | **1949** | **1698** | **1691** | **2127** | **1785** |
| **Manganese (Mn)** | mg kg⁻¹ | **99.0 b** | **368.5 a** | **40.8 B** | **41.9 B** | **794.3 A** | **974.5 A** |
| **Molybdenum (Mo)** | mg kg⁻¹ | **4.22 b** | **5.29 a** | **4.29** | **4.70** | **5.46** | **4.51** |

No significant Site × Plant species interaction was detected for the parameters shown. Scheffé post hoc tests were applied to significant main effects only (*p <* 0.05). Within each row, different lower-case letters indicate significant differences between Sites, whereas different upper-case letters indicate significant differences among Plant species.
