# Supplementary material for: Root-associated fungi in acid mine drainage-impacted environments
Source: Front Microbiol. 2026 Jun 10;17:1812818. doi: 10.3389/fmicb.2026.1812818 (PMC13293307; doi:10.3389/fmicb.2026.1812818)
Supplement: Supplementary file 5 [file table_5.docx]

Supplementary Table S5A. Main effects of site on root and shoot bioconcentration (RBCF, SBCF) and translocation factors

| Element | RBCF | | SBCF | | TF | |
| --- | --- | --- | --- | --- | --- | --- |
|  | **AMD-impacted site** | **Non-AMD-impacted site** | **AMD-impacted site** | **Non-AMD-impacted site** | **AMD-impacted site** | **Non-AMD-impacted site** |
| **Phosphorus (P)** | 0.95 b | 1.68 a | 0.90 | 1.22 | 1.09 | 0.95 |
| **Potassium (K)** | 4.3 10^-5^ | 3.1 10^-5^ | 3.8 10^-5^ | 2.7 10^-5^ | 0.90 | 1.22 |
| **Calcium (Ca)** | 2.8 10^-5^ | 2.8 10^-5^ | 4.1 10^-5^ | 3.2 10^-5^ | 1.54 | 1.36 |
| **Sulfur (S)** | 3.4 10^-5^ b | 2.8 10^-4^ a | 2.9 10^-5^ b | 2.8 10^-3^ a | 0.83 | 1.76 |
| **Copper (Cu)** | 0.23 | 0.36 | 0.08 b | 0.38 a | 0.47 b | 1.15 a |
| **Zinc (Zn)** | 0.53 | 0.57 | 0.63 | 0.57 | 1.62 | 1.21 |
| **Iron (Fe)** | 0.08 | 0.09 | 0.01 | 0.01 | 0.20 | 0.14 |
| **Aluminum (Al)** | 0.09 | 0.08 | 0.08 a | 0.04 b | 0.85 a | 0.50 b |
| **Manganese (Mn)** | 0.18 | 0.27 | 0.17 b | 0.33 a | 0.93 | 1.29 |
| **Molybdenum (Mo)** | 0.83 b | 1.34 a | 0.86 b | 1.82 a | 1.03 b | 1.44 a |

No significant Site × Plant species interaction was detected for the parameters shown. Scheffé post hoc tests were applied to significant Site main effects only (*p <* 0.05). Within each row, different lower-case letters indicate significant differences between Sites.
