# Supplementary material for: Root-associated fungi in acid mine drainage-impacted environments
Source: Front Microbiol. 2026 Jun 10;17:1812818. doi: 10.3389/fmicb.2026.1812818 (PMC13293307; doi:10.3389/fmicb.2026.1812818)
Supplement: Supplementary file 6 [file table_6.docx]

Table S6. Performance of the final RDA model (R^2^, adjusted R^2^ value, variance explained by constrained axes).

| Metric | Value |
| --- | --- |
| R^2^ (raw) | 0.9171 |
| R^2^ (adjusted) | 0.6327 |
| RDA1 explained variance (%) | 20.38 |
| RDA2 explained variance (%) | 11.81 |
