# Supplementary material for: Root-associated fungi in acid mine drainage-impacted environments
Source: Front Microbiol. 2026 Jun 10;17:1812818. doi: 10.3389/fmicb.2026.1812818 (PMC13293307; doi:10.3389/fmicb.2026.1812818)
Supplement: Supplementary file 7 [file table_7.docx]

Table S7. Variables included in the final RDA model, their marginal effects (variance explained, F, p-values) and RDA1 and RDA2 coefficients.

| Variable | Variance | F value | P value | RDA1 | RDA2 |
| --- | --- | --- | --- | --- | --- |
| Ca | 0.0342 | 3.9768 | 0.0001 | 0.2917 | 0.2709 |
| KTF | 0.0434 | 5.0376 | 0.0001 | 0.3035 | 0.3925 |
| P | 0.0274 | 3.1872 | 0.0001 | -0.1266 | -0.0627 |
| Cr | 0.0251 | 2.9152 | 0.0002 | 0.1818 | -0.1902 |
| ZnTF | 0.0295 | 3.4275 | 0.0003 | 0.0649 | -0.0884 |
| Cu | 0.0305 | 3.5403 | 0.0005 | -0.6935 | 0.3411 |
| SP | 0.0247 | 2.8661 | 0.0010 | 0.4269 | -0.2539 |
| Co | 0.0267 | 3.0986 | 0.0012 | -0.7108 | -0.0181 |
| SoilWC | 0.0240 | 2.7838 | 0.0021 | -0.3860 | -0.0129 |
| EC | 0.0233 | 2.7120 | 0.0022 | -0.3390 | -0.0475 |
| KRBCF | 0.0235 | 2.7348 | 0.0024 | -0.1124 | -0.1092 |
| RMo | 0.0231 | 2.6843 | 0.0024 | 0.0125 | 0.1684 |
| SZn | 0.0235 | 2.7275 | 0.0024 | 0.1637 | 0.1096 |
| CuTF | 0.0231 | 2.6870 | 0.0026 | 0.3347 | -0.0454 |
| SMo | 0.0227 | 2.6349 | 0.0032 | 0.2745 | -0.0673 |
| AlSBCF | 0.0228 | 2.6506 | 0.0033 | -0.2685 | -0.0177 |
| PSBCF | 0.0202 | 2.3516 | 0.0045 | 0.4684 | -0.2235 |
| S | 0.0202 | 2.3468 | 0.0072 | -0.5369 | -0.0301 |
| AlTF | 0.0185 | 2.1515 | 0.0153 | -0.1450 | 0.0695 |
| ZnSBCF | 0.0180 | 2.0927 | 0.0176 | 0.0881 | -0.0161 |
| As | 0.0170 | 1.9769 | 0.0245 | -0.4042 | 0.0869 |
| MnTF | 0.0149 | 1.7300 | 0.0512 | -0.1855 | 0.0214 |
| RK | 0.0144 | 1.6672 | 0.0634 | -0.1579 | -0.0808 |
| SMn | 0.0141 | 1.6417 | 0.0713 | 0.2454 | -0.0070 |
